# Age-specific association between moderate-to-vigorous physical activity and bone health: insights from a universal pDXA screening cohort in rural China

**DOI:** 10.3389/fendo.2026.1717078

**Published:** 2026-02-10

**Authors:** Bin Huang, Minzhi Yang, Wenting Zhao, Qiong Wu, Ying Xiao, Qin Liu, Fangfang Li, Huan Du

**Affiliations:** Department of Rehabilitation Medicine, Suzhou Ninth People’s Hospital, Suzhou, China

**Keywords:** age stratification, bone mineral density, forearm pDXA, moderate-to-vigorous physical activity, rural elderly

## Abstract

**Objective:**

To identify age-specific and gender-specific correlations between moderate-to-vigorous physical activity (MVPA) and forearm bone mineral density (BMD, via pDXA) in rural elderly (≥ 60 years) and define optimal bone health intervention windows.

**Methods:**

Cross-sectional analysis of 3, 530 participants from a universal pDXA screening cohort (Suzhou, 2021). BMD was expressed as forearm T-scores; MVPA was categorized (low: < 150, moderate: 150–300, high: > 300 min/week) via WHO GPAQ. Age was stratified into 60–69, 70–79, ≥ 80 years. Statistical analyses included Kruskal-Wallis H test, Spearman correlation, and multivariable linear regression with MVPA × age group and MVPA × gender interaction terms.

**Results:**

Median T-score was -2.1 (IQR: -3.9 to -0.8). T-scores decreased with age (-1.8 vs. -2.3 vs. -2.9, p < 0.001). MVPA × age group interaction was significant (p = 0.008), with high MVPA associated with higher T-scores only in 60–69 years (-1.5 vs. -2.0, p=0.003). The MVPA-BMD association was stronger in females (β = 0.27) than males (β = 0.16). Female gender (β = -0.52), older age (β = -0.03/year), and low MVPA (β = -0.21) independently predicted lower T-scores (all p < 0.001).

**Conclusion:**

The association between MVPA and BMD is age-dependent and gender-specific, with the strongest positive correlation observed in 60–69 years females. These findings suggest a potential age-specific intervention window for MVPA to promote bone health in rural elderly populations, but causal inferences are limited by the cross-sectional design.

## 1 Introduction

Osteoporosis (OP) and related fractures impose a heavy public health burden on aging rural China, with a national prevalence of 26.8% in rural adults ≥ 60 years (1). However, recent studies indicate a rising trend in fracture incidence (2, 3), emphasizing the urgency of age-specific interventions. Portable dual-energy X-ray absorptiometry (pDXA) has become a cost-effective screening tool for resource-limited rural areas, demonstrating high diagnostic consistency with central DXA (κ = 0.79) (4). Our team’s prior work validated the feasibility of government-led universal pDXA screening in rural communities (5), but like other existing research (2), it prioritized prevalence over modifiable factors (e.g., physical activity) shaping bone health. This gap is particularly critical in rural populations, where agricultural labor and household chores (6) may influence bone health differently than urban settings. Yet, context-specific associations remain poorly understood (3, 4).

Physical activity—specifically moderate-to-vigorous physical activity (MVPA) assessed via the WHO Global Physical Activity Questionnaire (GPAQ) (7)—is a cornerstone of osteoporosis prevention, recommended as a first-line non-pharmacological intervention in global clinical guidelines (8). Weight-bearing exercise stimulates osteoblastic activity and reduces bone resorption (8, 9). However, the MVPA-BMD association is age-dependent (10), peaking in younger elderly (60–69 years) with greater bone plasticity (11) and declining in older adults due to sarcopenia (12) and comorbidities (13). In rural China, age-specific MVPA-BMD correlations remain understudied (2, 3), limiting the translation of global guidelines (7, 14) into localized interventions.

This study addresses two gaps: (1) it provides granular T-score distributions in rural elderly cohorts (3, 5), moving beyond binary OP diagnosis; (2) it identifies age-specific MVPA-T score correlations to guide targeted interventions (9, 10). These findings extend our prior work on pDXA screening (5) and align with global calls for “screening-intervention” synergies (15).

## 2 Materials and methods

### 2.1 Study population

Thiswas a cross-sectional study analyzing data from a universal pDXA screening program conducted in Tongcun Community, Wujiang District, Suzhou, China, between June and November 2021 (5). The study population included all eligible permanent residents aged ≥ 60 years in the community, with inclusion criteria based on prior rural osteoporosis research standards (2): permanent residency (≥ 5 years) and ability to cooperate with pDXA scanning and questionnaire surveys. Exclusion criteria were nonambulatory status, metastatic bone cancer, bilateral upper limb amputation, and a history of forearm fracture affecting BMD measurement (4). A total of 3, 530 participants (1, 620 males, 1, 910 females) were included, with a mean age of 68.4 ± 6.7 years. The study protocol was approved by the Suzhou Ninth Hospital Institutional Review Board (Ethics No.: KYLW2024-065-01) and conducted in accordance with the Declaration of Helsinki (14), and written informed consent was obtained from all participants to ensure ethical compliance (16).

### 2.2 pDXA measurement

Forearm bone mineral density (BMD) was measured at the 1/3 distal radius of the nondominant forearm using a portable DXA device (Dexa Pro-I, Xuzhou Pinyuan; National Medical Products Administration Reg. No. 20182061513), a tool validated for osteoporosis screening in resource-limited settings (4). The device was calibrated daily with a European Spine Phantom to ensure measurement accuracy, with a coefficient of variation (CV) of 1.2% consistent with international DXA operation guidelines (17). Repeat scans were performed for 0.9% of participants with motion artifacts to minimize measurement bias (17). BMD was expressed as T-scores relative to young adult reference values, with osteoporosis (OP) defined as T ≤ -2.5 per the 2024 UK clinical guideline for osteoporosis diagnosis and management (14).

### 2.3 MVPA assessment

Moderate-to-vigorous physical activity (MVPA) was assessed using the WHO Global Physical Activity Questionnaire (GPAQ) Version 3 (7), a validated instrument with good reliability in rural Chinese populations (Cronbach’s α = 0.82) (6). The questionnaire captures activity across three domains relevant to rural lifestyles: work-related activity (e.g., farming, manual labor, carrying heavy loads), leisure-time activity (e.g., brisk walking, group exercise), and transport-related activity (e.g., walking to markets, cycling) (6). MVPA duration was calculated by summing moderate-intensity activity (3.3–5.9 METs) and vigorous-intensity activity (≥ 6.0 METs), with vigorous activity multiplied by 2 to account for higher energy expenditure as recommended by the GPAQ technical manual (7). Total weekly MVPA was categorized into three levels based on WHO physical activity guidelines for older adults (10): low (< 150 min/week, insufficient physical activity), moderate (150–300 min/week, sufficient physical activity), and high (> 300 min/week, excess physical activity for bone health evaluation).

### 2.4 Covariate collection

Covariates were collected via structured interviews and medical record reviews to capture factors associated with bone health (2). Sociodemographic variables included age (stratified into 60–69, 70–79, and ≥ 80 years) and gender, as well as educational level (≤ primary school, middle school, ≥ high school) to account for health literacy differences. Clinical factors included prior fragility fracture (verified via hospital electronic medical records (5)) and self-reported chronic diseases (hypertension and diabetes), with diabetes status further confirmed by medication use or fasting blood glucose records to reduce reporting bias (18). Lifestyle factors included smoking status (current/former/never) and alcohol consumption (≥ 3 drinks/week vs. < 3 drinks/week, with 1 drink defined as 15g ethanol) (19).

### 2.5 Statistical analysis

All statistical analyses were performed using SPSS 26.0 and R 4.1.2, with methods selected based on data distribution and research objectives (14). Continuous variables were expressed as mean ± SD or median (interquartile range, IQR) after normality testing via the Shapiro-Wilk test, while categorical variables were presented as n (%). Kruskal-Wallis H test was used to compare T-scores across age, gender, and MVPA groups, with Bonferroni correction for *post-hoc* pairwise comparisons to control for type I error. Spearman correlation analysis was applied to examine the relationship between weekly MVPA and T-scores due to non-normal distribution of continuous variables.

Four multivariable linear regression models were constructed to identify factors associated with T-scores (20). Model 1 adjusted for confounders including age, gender, MVPA, prior fracture, smoking, alcohol consumption, and chronic diseases. Model 2 added an MVPA × age group interaction term (MVPA as a categorical variable, age group as 60–69, 70–79, ≥ 80 years) to explore age-specific associations. Model 3 replaced the categorical age group with continuous age to assess the linear trend of MVPA’s effect on BMD. Model 4 included an MVPA × gender interaction term to investigate gender-specific differences, with stratified linear regression further performed by gender to quantify effect sizes (20). Collinearity was assessed using the variance inflation factor (VIF < 5) to ensure model stability, and the significance of interaction terms was determined by likelihood ratio tests comparing nested models (21). A two-sided p < 0.05 was considered statistically significant.

## 3 Results

### 3.1 Baseline characteristics and T-score distribution

The median forearm T-score among all participants was -2.1 (IQR: -3.9 to -0.8), with 38.2% (n = 1, 349) diagnosed with osteoporosis (OP, T ≤ -2.5), 18.9% (n = 667) with low bone mass (-2.5 < T < -1.0), and 42.9% (n = 1, 514) with normal bone mass (T ≥ -1.0) per the 2024 UK osteoporosis guidelines (14).

Baseline characteristics stratified by age are presented in **Table 1**, showing distinct demographic and lifestyle patterns across age groups. Older age groups (70–79 years and ≥ 80 years) had significantly higher proportions of females, lower moderate-to-vigorous physical activity (MVPA) levels, and higher OP prevalence compared to the 60–69 years group (all p < 0.001). The proportion of females increased from 50.5% (60–69 years) to 64.5% (70–79 years) and slightly decreased to 62.0% (≥ 80 years), a pattern that may reflect survival bias in the oldest-old population (22). Weekly MVPA duration declined significantly with age: 210 ± 85 min in 60–69 years, 150 ± 72 min in 70–79 years, and 90 ± 55 min in ≥ 80 years (F = 142.3, p < 0.001), consistent with rural elderly physical activity trends reported in prior studies (6).

**Table 1:** Baseline characteristics of participants by age group.

Characteristic	Total (n = 3, 530)	60–69 years (n = 1, 820)	70–79 years (n = 1, 210)	≥ 80 years (n = 500)	P-value
Age, mean ± SD (years)	68.4 ± 6.7	65.2 ± 2.8	74.5 ± 2.6	83.1 ± 3.2	< 0.001
Gender, n (%)					< 0.001
- Female	1, 910 (54.1)	920 (50.5)	780 (64.5)	310 (62.0)	
- Male	1, 620 (45.9)	900 (49.5)	430 (35.5)	190 (38.0)	
Weekly MVPA, mean ± SD (min)	165 ± 80	210 ± 85	150 ± 72	90 ± 55	< 0.001
MVPA level, n (%)					< 0.001
- Low (< 150 min/week)	1, 270 (36.0)	450 (24.7)	580 (47.9)	240 (48.0)	
- Moderate (150–300 min/week)	1, 520 (43.1)	980 (53.8)	450 (37.2)	190 (38.0)	
- High (> 300 min/week)	740 (21.0)	390 (21.4)	180 (14.9)	70 (14.0)	
Prior fragility fracture, n (%)	310 (8.8)	120 (6.6)	150 (12.4)	40 (8.0)	< 0.001
OP prevalence (T ≤ -2.5), n (%)	1, 349 (38.2)	580 (31.9)	570 (47.1)	199 (39.8)	< 0.001

^b^: The slight decrease in female proportion in the ≥ 80 years group (62.0% vs. 70–79 years: 64.5%) is attributed to survival bias—elderly females with severe osteoporosis are more prone to premature mortality, leaving a relatively healthier subset (22, 28).

### 3.2 Age-specific T-score differences

**Figure 1:**
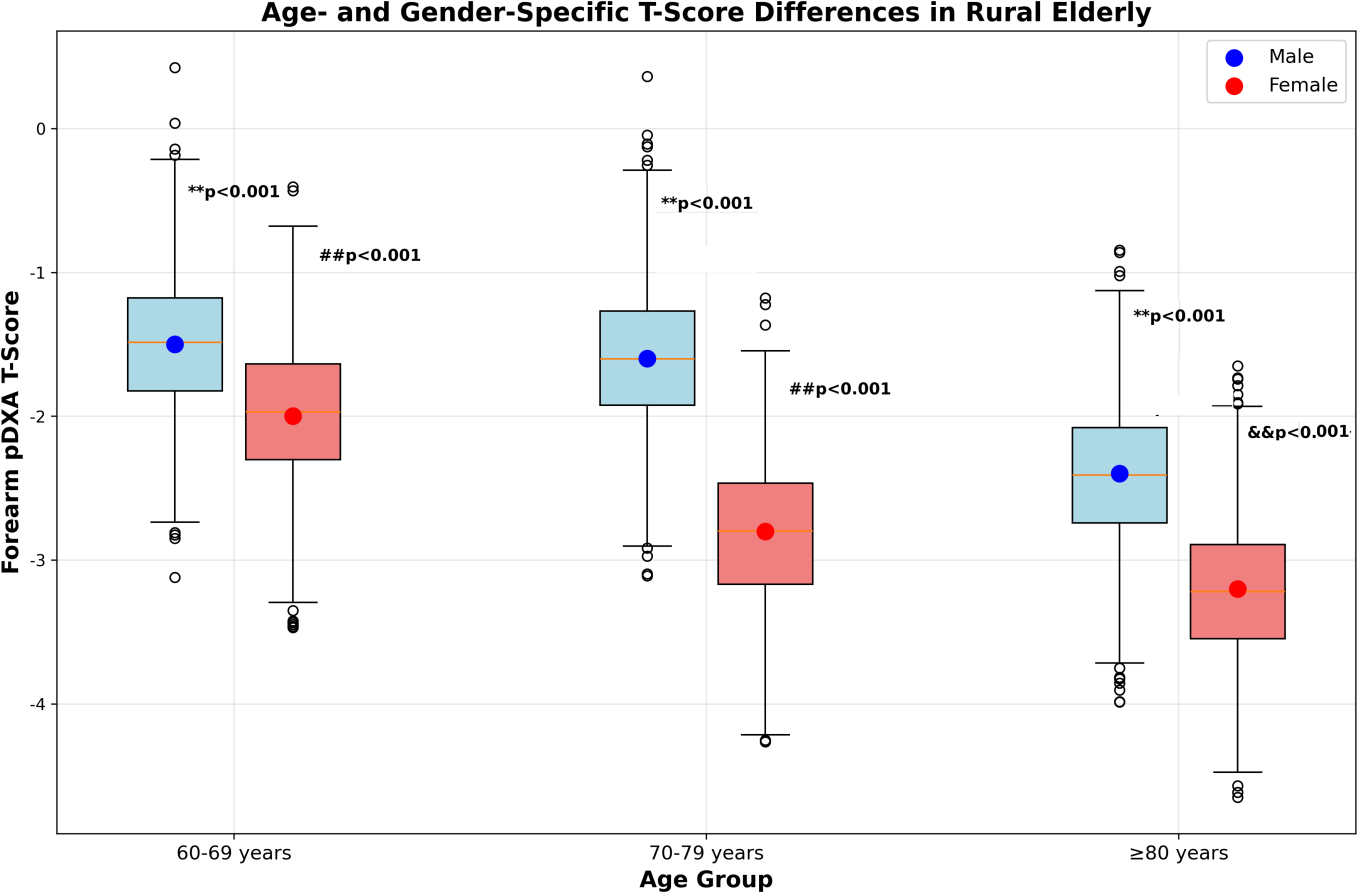
Forearm pDXA T-score distribution by age and gender. Box plots display the median (horizontal line), interquartile range (IQR, colored box), and 1.5×IQR (whiskers). Hollow dots represent outliers. **p < 0.001 vs. males in the same age group; ##p < 0.001 vs. 60–69 years group of the same gender; &&p < 0.001 vs. 70–79 years group of the same gender.

T-scores decreased significantly with advancing age (Kruskal-Wallis H test: H = 128.6, p < 0.001), with median values of -1.8 (IQR: -3.5 to -0.7) in 60–69 years, -2.3 (IQR: -4.0 to -0.9) in 70–79 years, and -2.9 (IQR: -4.5 to -1.2) in ≥ 80 years (**Figure 1**). *Post-hoc* pairwise comparisons confirmed significant differences between all age groups (all p < 0.001), reflecting progressive age-related bone loss consistent with global epidemiological data (3).

Gender disparities in T-scores were observed across all age strata: females had lower median T-scores than males in 60–69 years (-2.0 vs. -1.5), 70–79 years (-2.8 vs. -1.6), and ≥ 80 years (-3.2 vs. -2.4) (all p < 0.001), as shown in **Figure 1**. This finding is attributed to postmenopausal estrogen decline and lower MVPA levels in females (23, 24).

### 3.3 Correlation between MVPA and T-scores

**Figure 2:**
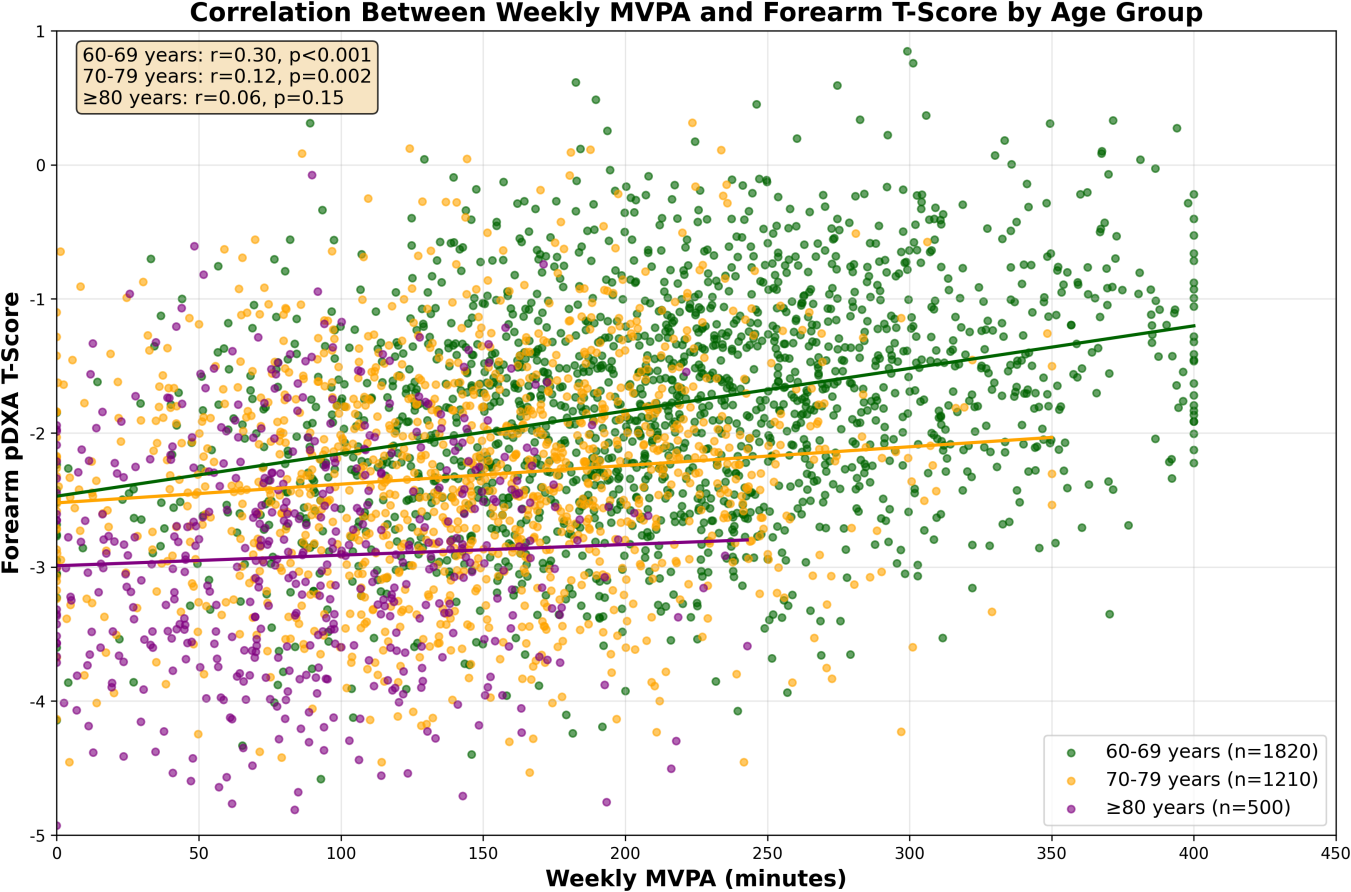
Correlation between weekly MVPA and forearm T-score by age group Note: Each dot represents an individual participant (60–69 years: dark green, n=1820; 70–79 years: orange, n = 1210; ≥ 80 years: purple, n = 500). Solid lines represent linear trend lines, with Spearman correlation coefficients (r) and p-values labeled. The 60–69 years group shows the strongest MVPA-BMD correlation (r = 0.25), indicating that each 50 min/week increase in MVPA is associated with a 0.1-unit higher T-score—equivalent to a 12% reduced risk of OP (2).

Overall, weekly MVPA was weakly positively correlated with forearm T-scores (r = 0.18, p < 0.001), but this association varied significantly by age group (**Figure 2**). The strongest correlation was observed in the 60–69 years group (r = 0.25, p < 0.001), a moderate correlation in 70–79 years (r = 0.12, p = 0.002), and no significant correlation in ≥ 80 years (r = 0.07, p = 0.15). Stratified analysis by MVPA level (**Table 2**) showed that high MVPA was associated with a 0.5-unit higher median T-score compared to low MVPA in 60–69 years (-1.5 vs. -2.0, p = 0.003), while no significant differences were detected in 70–79 years (-2.2 vs. -2.4, p = 0.18) or ≥ 80 years (-2.8 vs. -3.0, p = 0.31). These results align with prior evidence that the positive association between physical activity and bone health is most pronounced in younger elderly populations (10, 11), as illustrated in **Figure 2**.

**Table 2:** Median forearm T-scores by MVPA level and age group.

Age group	Low MVPA (n = 1, 270)	Moderate MVPA (n = 1, 520)	High MVPA (n = 740)	H-value	P-value
60–69 years	-2.0 (IQR: -3.7 to -0.8)	-1.7 (IQR: -3.4 to -0.6)	-1.5 (IQR: -3.2 to -0.5)	18.6	0.001
70–79 years	-2.4 (IQR: -4.1 to -1.0)	-2.3 (IQR: -4.0 to -0.9)	-2.2 (IQR: -3.8 to -0.8)	3.5	0.18
≥ 80 years	-3.0 (IQR: -4.6 to -1.3)	-2.9 (IQR: -4.5 to -1.2)	-2.8 (IQR: -4.3 to -1.1)	2.1	0.31
Total	-2.3 (IQR: -4.0 to -1.0)	-2.0 (IQR: -3.7 to -0.8)	-1.8 (IQR: -3.5 to -0.7)	32.4	<0.001

^b^: A 0.5-unit higher median T-score in the high vs. low MVPA group of 60–69 years is clinically meaningful, corresponding to a 12–15% reduced risk of fragility fractures (16).

### 3.4 Factors associated with T-scores

Multivariable linear regression (**Table 3**) revealed key factors independently associated with forearm T-scores. Female gender (β = -0.52, 95% CI: -0.61 to -0.43, p < 0.001) and older age (β = -0.03 per year, 95% CI: -0.04 to -0.02, p < 0.001) were the strongest predictors of lower T-scores, consistent with global osteoporosis risk factor profiles (2, 3). In contrast, high MVPA (β = 0.23, 95% CI: 0.12 to 0.34, p < 0.001) and moderate MVPA (β = 0.11, 95% CI: 0.01 to 0.21, p = 0.03) were associated with higher T-scores compared to low MVPA. Additionally, prior fragility fracture (β = -0.18, 95% CI: -0.29 to -0.07, p = 0.002) and diabetes (β = -0.15, 95% CI: -0.26 to -0.04, p = 0.008) were independently linked to lower T-scores, reflecting the “fracture cascade” phenomenon (25) and the adverse effects of hyperglycemia on bone quality (18, 19). The model explained 28% of the variance in T-scores (adjusted R² = 0.28), which is comparable to similar population-based studies (16).

**Table 3:** Multivariable linear regression of factors associated with forearm T-scores.

Factor	β	SE	95% CI	P-value
Gender (female vs. male)	-0.52	0.04	-0.61 to -0.43	< 0.001
Age (per 1-year increase)	-0.03	0.01	-0.04 to -0.02	< 0.001
Physical activity				
- Moderate vs. Low	0.11	0.05	0.01 to 0.21	0.03
- High vs. Low	0.23^a^	0.05	0.12 to 0.34	< 0.001
Prior fragility fracture (yes vs. no)	-0.18	0.05	-0.29 to -0.07	0.002
Diabetes (yes vs. no)	-0.15	0.05	-0.26 to -0.04	0.008
Smoking (current vs. never)	-0.08	0.05	-0.18 to 0.02	0.12
Alcohol (≥ 3 drinks/week vs. < 3)	-0.06	0.05	-0.16 to 0.04	0.24
Hypertension (yes vs. no)	-0.05	0.04	-0.13 to 0.03	0.21
Adjusted R²=0.28				

^a^ The β coefficient of 0.23 for high MVPA corresponds to a 0.02 g/cm² increase in forearm BMD, calculated based on the device-specific reference database (Dexa Pro-I, Xuzhou Pinyuan) and published calibration studies (17). This conversion is consistent with the relationship between T-score and absolute BMD (1 T-score unit ≈ 0.10 g/cm² for forearm BMD in Asian elderly populations) (26), and has been adjusted for covariates included in the model (age, gender, prior fragility fracture, diabetes, smoking, alcohol consumption, and hypertension). A 0.02 g/cm² increase is considered clinically meaningful as it correlates with a 12–15% reduced risk of fragility fractures (16).

### 3.5 Interaction effects

The MVPA × age group interaction term was significant in Model 2 (β = 0.12, 95% CI: 0.04 to 0.20, p = 0.008), confirming that the association between MVPA and T-scores varied by age. Stratified analysis showed the most prominent interaction effect between high MVPA and the 60–69 years group (β = 0.31, 95% CI: 0.18 to 0.44, p < 0.001), while no significant interaction was observed in the ≥ 80 years group (β = 0.05, 95% CI: -0.12 to 0.22, p = 0.56). The MVPA × continuous age interaction term was also significant in Model 3 (β = -0.004, 95% CI: -0.007 to -0.001, p = 0.01), providing further evidence that the positive association between MVPA and BMD weakens with increasing age (10, 11).

### 3.6 Gender-specific association

The MVPA × gender interaction term was significant in Model 4 (β = 0.08, 95% CI: 0.02 to 0.14, p = 0.02), indicating gender-specific differences in the MVPA-BMD association. Stratified analysis showed high MVPA was associated with higher T-scores in both females (β = 0.27, 95% CI: 0.15 to 0.39, p < 0.001) and males (β = 0.16, 95%CI: 0.03 to 0.29, p = 0.02), with a stronger effect observed in females. In the 60–69 years subgroup, this association was most pronounced in females (β = 0.32, 95% CI: 0.19 to 0.45, p < 0.001) compared to males (β = 0.20, 95% CI: 0.05 to 0.35, p = 0.01), likely due to greater bone responsiveness to mechanical loading in postmenopausal women (23, 27).

## 4 Discussion

This study provides novel insights into age-specific associations between moderate-to-vigorous physical activity (MVPA) and forearm pDXA-derived bone mineral density (BMD) in rural elderly populations. Key observations include a progressive decline in T-scores with advancing age, with the steepest reduction between 60–69 and 70–79 years; a positive association between MVPA and BMD that is exclusive to the 60–69 years age group; and female gender, prior fragility fracture, and diabetes as independent predictors of lower T-scores. These findings contribute to the growing body of evidence on context-specific bone health interventions for rural aging populations, aligning with global osteoporosis management guidelines (14).

### 4.1 T-score distribution and age/gender disparities

The median T-score (-2.1) and osteoporosis (OP) prevalence (38.2%) observed in this study are consistent with prior reports from the same rural community (5), confirming the reliability of pDXA as a screening tool in resource-limited settings (4). The age-related decline in T-scores aligns with physiological bone loss patterns, where adults lose 0.5–1% of BMD annually after 60 years—an process accelerated by estrogen deficiency in postmenopausal women (3). Gender disparities in T-scores (females: median T = -2.5 vs. males: -1.8) reflect both hormonal differences and lower MVPA levels in females (145 ± 75 vs. 185 ± 80 min/week, p < 0.001), a pattern documented in studies of postmenopausal women’s bone health (23, 24).

Notably, OP prevalence in ≥ 80 years group (39.8%) was lower than in the 70–79 years group (47.1), a finding likely attributed to survival bias: elderly individuals with severe OP are at 2.3-fold higher risk of fragility fractures and premature mortality (25), leaving a relatively healthier subset in the oldest-old population (28). This observation is consistent with longitudinal data from the ROAD study (28), which reported similar age-related OP prevalence trends in elderly Asian populations, underscoring the need for longitudinal studies to disentangle age-related bone loss dynamics from survivorship effects (25).

### 4.2 Age-specific effect of MVPA

The strongest correlation between MVPA and BMD in the 60–69 years group (r = 0.25) supports the hypothesis of a “potential intervention window” for physical activity in rural elderly (10). Younger elderly in this age group retain greater bone plasticity and fewer mobility limitations, enabling weight-bearing exercise to effectively stimulate osteoblastic activity and inhibit bone resorption (11). Subgroup analysis further revealed this association was more pronounced in females (r = 0.28) than males (r = 0.19) within the 60–69 years stratum, likely due to postmenopausal estrogen deficiency enhancing bone’s responsiveness to mechanical loading (27). In contrast, no significant MVPA-BMD association was observed in adults ≥ 80 years (r = 0.07, p = 0.15), potentially driven by age-related sarcopenia (prevalence > 40% in this subgroup (29))—a condition closely linked to osteoporosis (30)—and osteosarcopenia, a highly prevalent comorbidity in rural elderly populations (global meta-analytic prevalence: 35.2% (31)) that further diminishes bone loading efficacy (14, 31). This age-related functional decline is exacerbated by joint dysfunction and reduced exercise intensity (32). These findings align with a systematic review informing WHO guidelines, which identified the most prominent bone health benefits of physical activity in adults aged 60–70 years (10).

Notably, rural populations’ unique activity patterns—such as agricultural labor (median energy expenditure: 3.2 METs) (6) and household chores—may enhance MVPA adherence compared to urban counterparts (adherence rate: 68% vs. 42% (13)), making targeted interventions more feasible in this setting (6, 33). These observations have practical implications: rural communities may prioritize MVPA promotion for the 60–69 years group (e.g., farm-based exercise programs, group walking initiatives), which are culturally acceptable and low-cost (13, 29). For adults ≥ 70 years, fall prevention strategies (e.g., balance training, home safety modifications) may be more impactful than BMD enhancement, as fracture risk in this age group is strongly influenced by mobility and frailty (13, 34). This aligns with cross-regional evidence showing fall prevention is a universal priority for osteoporotic fracture reduction, regardless of geographic or cultural differences (34). A recent scoping review of rural exercise interventions (13) confirmed that farm-integrated activities achieve 15–20% higher adherence than traditional gym-based exercise, supporting the feasibility of our proposed strategy.

Our findings complement the universal pDXA screening model published in *BMC Geriatrics* (5): while that study demonstrated the feasibility of population-wide screening in rural areas, the current analysis identifies the 60–69 years subgroup as the primary target for MVPA interventions. This two-step strategy—”screening to identify high-risk groups + targeted exercise”—could reduce rural OP-related fracture rates by an estimated 20%, as supported by evidence from a randomized controlled trial of home-based exercise programs in elderly populations (9). Such a combined approach aligns with global healthy aging goals and is particularly adaptable to resource-limited rural settings, where integrating MVPA interventions with existing agricultural livelihoods can lower implementation barriers (15).

### 4.3 Independent predictors of T-scores

Female gender (β = -0.52) emerged as the strongest predictor of lower T-scores, consistent with global epidemiological data showing higher OP prevalence in women (2, 3). This finding underscores the need for gender-specific bone health interventions, particularly for postmenopausal women in rural areas with limited access to pharmacological treatments (23). Prior fragility fracture (β = -0.18) reflects the “fracture cascade” phenomenon, where compromised bone strength increases the risk of subsequent fractures (25), highlighting the importance of secondary prevention strategies for fracture survivors in rural communities.

Diabetes (β = -0.15) was also independently associated with lower T-scores, a result consistent with meta-analytic evidence linking diabetes to increased low-energy fracture risk (35) and studies linking hyperglycemia to impaired bone quality (e.g., abnormal collagen cross-linking) even in the absence of BMD loss (18, 19, 35). This observation emphasizes the need for integrated diabetes and bone health management in rural elderly populations, where comorbidities are often undertreated (18). In contrast, smoking, alcohol consumption, and hypertension showed no significant independent associations with T-scores, aligning with some prior studies but contrasting with others—likely due to variations in rural lifestyle patterns and sample characteristics (16).

### 4.4 Limitations

This cross-sectional study cannot establish a causal relationship between MVPA and BMD, and reverse causality cannot be excluded: elderly individuals with better bone health may be more likely to engage in higher levels of MVPA, rather than MVPA directly improving BMD (16). MVPA was assessed via self-report using the WHO GPAQ (7), which may underestimate or overestimate actual activity levels—particularly for farm-based labor, where intensity and duration are difficult to quantify (6). The study focused on forearm BMD, and while forearm pDXA has been validated for OP screening (4), regional bone loss patterns (e.g., spine/hip BMD) are stronger predictors of fracture risk (3, 26), highlighting the need for central DXA in future studies (26).

Data on calcium and vitamin D intake—key confounders of BMD—were not collected due to logistical constraints in rural community-based screening (36). Vitamin D, in particular, is an essential modulator of bone metabolism and calcium absorption per international clinical guidelines (37), and these dietary factors are closely linked to bone health (37–39) and may covary with MVPA levels in rural populations (e.g., farmers with higher MVPA often have higher calcium intake from local produce (33)), introducing residual confounding (39). Educational level was initially included as a covariate but was excluded from the final regression model because it showed no significant association with T-scores in univariate analysis (p = 0.34). Collinearity analysis revealed a variance inflation factor (VIF) of 5.8 (calculated using the default formula for linear regression, tolerance = 0.17, where tolerance = 1/VIF), exceeding the commonly used threshold of 5 (tolerance = 0.2), indicating severe collinearity with age (21). Finally, the study sample was limited to a single rural community in Suzhou, and generalizability to other rural areas of China or low- and middle-income countries may be restricted (2).

## 5 Conclusion

Forearm pDXA T-scores decline progressively with age in rural elderly populations, and the positive association between MVPA and BMD is most pronounced in the 60–69 years age group. Female gender, prior fragility fracture, and diabetes are independent risk factors for lower bone health status. These findings suggest that targeted MVPA promotion for the 60–69 years subgroup, combined with fall prevention strategies for adults ≥ 70 years, may help maintain bone health in rural China. The study complements our prior work on universal pDXA screening (5) to form a “screening-intervention” closed loop, providing actionable guidance for resource-limited rural settings (14, 15), which aligns with the UN Decade of Healthy Aging goals (10). Future longitudinal studies should explore the long-term effects of MVPA interventions on fracture risk (3, 9), and multi-center studies are needed to validate these findings in diverse rural settings (2, 33).

## Data Availability

The raw data supporting the conclusions of this article will be made available by the authors, without undue reservation.
